# Derivatization of N-Acyl Glycines by 3-Nitrophenylhydrazine for Targeted Metabolomics Analysis and Their Application to the Study of Diabetes Progression in Mice

**DOI:** 10.1021/acs.analchem.2c02507

**Published:** 2023-01-19

**Authors:** Li Xiang, Yi Ru, Jingchun Shi, Li Wang, Hongzhi Zhao, Yu Huang, Zongwei Cai

**Affiliations:** † State Key Laboratory of Environmental and Biological Analysis, Department of Chemistry, Hong Kong Baptist University, Hong Kong 999077, China; ‡ Department of Biomedical Sciences, City University of Hong Kong, Hong Kong 999077, China; § Ministry of Education Key Laboratory of Pollution Processes and Environmental Criteria, College of Environmental Science and Engineering, Nankai University, Tianjin 300350, China

## Abstract

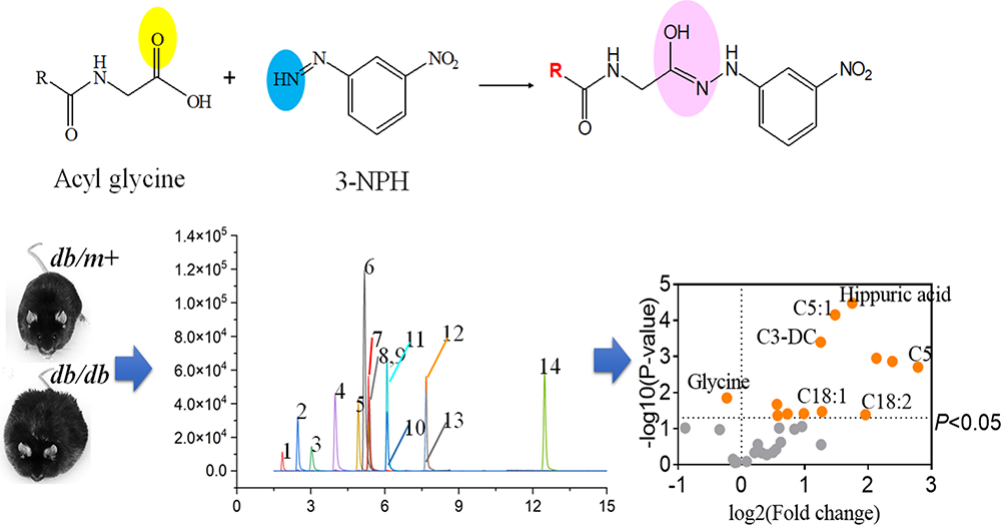

N-Acyl glycines (NAGlys) are an important class of metabolites in the detoxification system of the human body. They have been used in the diagnosis of several metabolic diseases. Liquid chromatography–mass spectrometry (LC–MS) is the most frequently used NAGlys detection platform. Here, we describe a simple and sensitive method of NAGlys detection by LC–MS in plasma and urine samples. This approach is based on the use of a derivatization reagent, 3-nitrophenylhydrazine. The reaction is quick in aqueous solution, and no quenching step is needed. To expand the coverage of NAGlys when standards are not available, NAGlys were first identified based on high-resolution LC–MS. Quantification was subsequently carried out on triple quadrupole LC–MS. This approach allowed a much broader measurement of NAGlys (41 NAGlys in total), especially when authentic standards are unavailable. Comprehensive analysis of NAGlys with this new method was applied in plasma and urine samples of *db/db* diabetic and non-diabetic *db*/*m+* control mice. The majority of detected NAGlys were altered with high differentiation ability in plasma and urine samples from diabetic and non-diabetic mice. These identified NAGlys hold the potential to be diagnostic biomarkers for type II diabetes and diabetic complications.

## Introduction

N-Acyl glycines (NAGlys) are a class of glycine conjugates generated during the detoxification of metabolites.^1,2^ In this process, glycine conjugates with various toxic metabolites, such as branched-chain amino acid (BCAA) metabolites, fatty acid oxidation intermediates, benzoate, and polyphenol metabolites, which might be toxic when they accumulate in an organism. Contrarily, the resulting NAGlys are less toxic, more hydrophilic, and can be excreted via urine. Altered levels of NAGlys in urine have been reported in kidney disease, obesity, and diabetes.^3,4^ Thus, identification and quantification of NAGlys potentially can be used for the diagnosis of metabolic diseases.

Gas chromatography–mass spectrometry (GC–MS) has been applied for the detection of NAGlys.^5,6^ Although GC–MS provides excellent separation performance, they are labor-intensive, and additional sample preparation steps are usually required. For instance, an additional derivatization step by utilizing methoxyamine to first form oxime products before derivatization with trimethylsilyl is usually needed,^7^ and quenching and purification steps are required by bis(trifluoromethyl)benzyl (BTFMB) derivatization.^5^ Consequently, it is time-consuming. In addition, compared with liquid chromatography coupled with mass spectrometry (LC–MS), the detection sensitivity by GC–MS is relatively lower.^8^

In addition to GC–MS analysis, LC–MS has also been applied for the analysis of NAGlys for a long time.^9,10^ Their direct detection in urine samples has been investigated.^11^ However, owing to the wide polarity of NAGlys, it is hard to quantify NAGlys with high and low polarity simultaneously and accurately. Derivatization has been proved to be effective in improving sensitivity and coverage. Acylation, such as methylation, acetylation, and *n*-butylation are frequently used.^8,12^ However, the reactions were usually limited to non-aqueous solutions, and additional drying and reconstitution steps of the derivatized samples are required, which is also relatively time-consuming. Stanislaus *et al*. developed a derivatization method using *p*-dimethylaminophenacyl (DmPA) bromid and achieved satisfactory sensitivity. Nevertheless, the reaction required a high-temperature condition (90 °C), and a quenching step was indispensable.^13^

**Figure 1:**
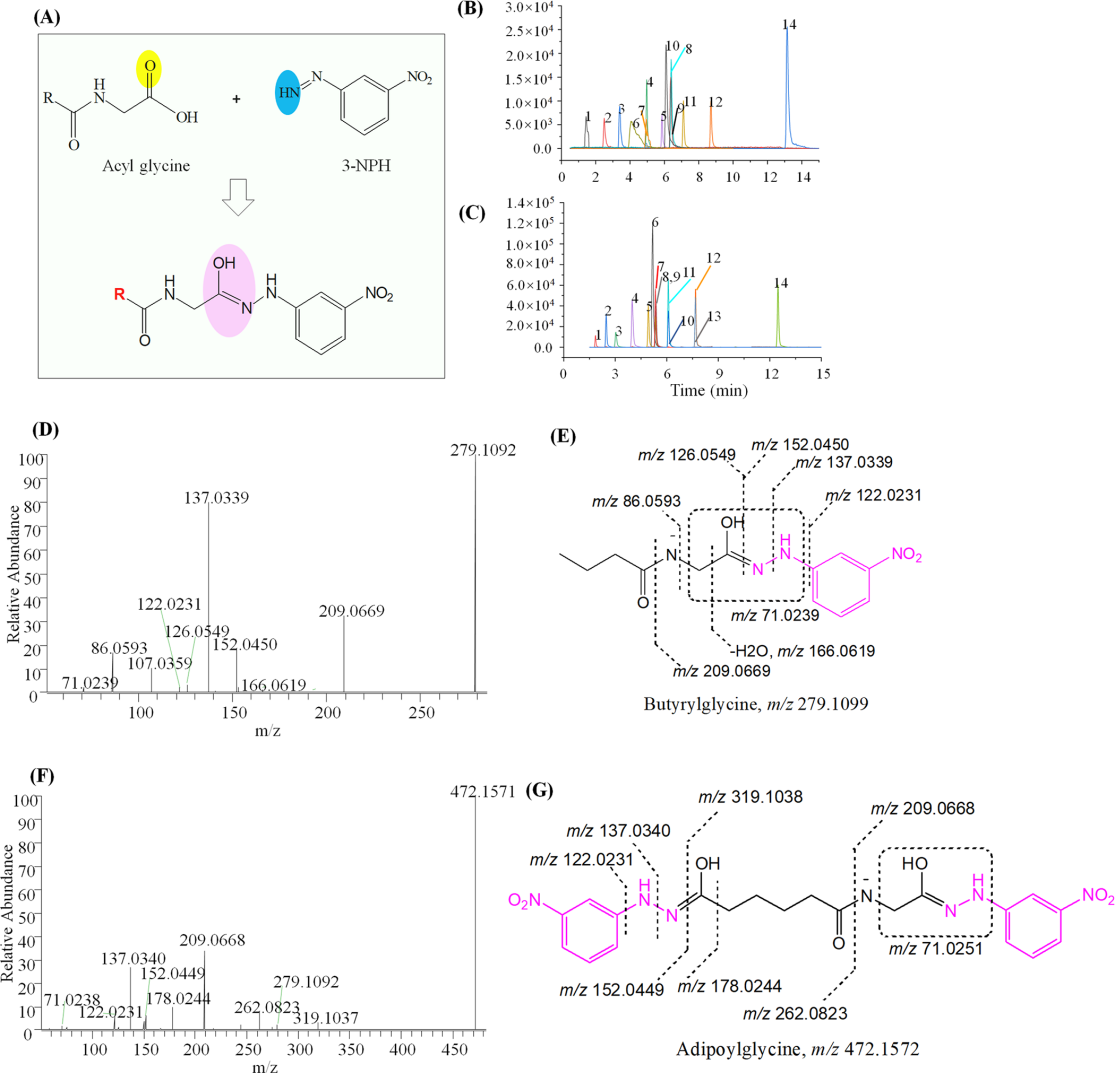
Proposed fragmentation patterns of 3-NPH-derivatized N-acyl glycine. (A) Schematic diagram of N-acyl glycine and 3-NPH conjugation. Representative N-acyl glycine chromatography (authentic standards) (B) without or (C) with 3-NPH derivatization. 1, glycine; 2, acetylglycine; 3, propionylglycine; 4, butyrylglycine; 5, isovalerylglycine; 6, glutarylglycine; 7, adipoylglycine; 8, hippuric acid; 9, hippuric acid-*d*5; 10, suberoylglycine; 11, hexanoylglycine; 12, octanoylglycine; 13, octanoylglycine-*d*2; 14, palmitoylglycine. The chromatography of NAGlys was generated from authentic standards. (D) MS/MS spectra of 3-NPH-derivatized butyrylglycine. (E) Proposed fragmentation patterns of butyrylglycine. (F) MS/MS spectra of 3-NPH-derivatized adipoylglycine. (G) Proposed fragmentation patterns of adipoylglycine.

3-Nitrophenylhydrazine (3-NPH) is a phenylhydrazine derivatizing reagent commonly used to derivatize carboxyl- and carbonyl-containing metabolites. Previous studies applied 3-NPH in deriving a broad range of metabolites, including carbohydrates, fatty acids, amino acids, indoles, benzenoids, bile acids, acylcarnitines, and phosphorylated metabolites, and obtained satisfactory quantification results.^14,15^ To the best of our knowledge, our study was the first attempt to apply the 3-NPH derivatization strategy to comprehensively detect NAGlys (Figure 1a) by LC–MS. The NAGlys derivatization procedure is more straightforward and can be conducted at a lower reaction temperature in aqueous solution. More importantly, the detection sensitivity is improved, and the coverage of NAGlys is expanded with a single injection by LC–MS detection in biofluids.

In this study, NAGlys in plasma and urine samples of *db/db* diabetic and *db/m+* control mice were derived by 3-NPH and subjected to LC–MS detection. The results show that the serum and urine NAGlys contents vary between *db/db* and *db/m+* mice. In particular, the content of NAGlys in urine is significantly correlated with the progress of diabetes, which could be used as early diagnostic biomarkers for diabetes. Our study suggested that the metabolic shifts of NAGlys might be a pathological event for the advancement of diabetes and diabetic complications.

## Experiments

### Materials and Reagents

HPLC-grade methanol was obtained from RIC Labscan Ltd. Co., (Bangkok, Thailand). HPLC-grade acetonitrile (ACN) was purchased from Tedia Company (Fairfield, OH, USA). 1-Ethyl [3-(dimethylamino)propyl]carbodiimide-HCl (EDCI, ≥98%) was obtained from Tokyo Chemical Industry (TCI, Japan). Formic acid (FA), 3-nitrophenylhydrazine, pyridine, and acyl glycine standards were purchased from Sigma Aldrich (St. Louis, MO, USA). Water was prepared from a Milli-Q Ultrapure Water System (Millipore, Billerica, MA, USA). Unless specified, all other reagents were of analytical grades.

### Animal Experiments and Sample Collection

In the current study, C57BL/KsJ-*db/db* male mice were chosen as type 2 diabetes mellitus (T2DM) model mice, and *db/m+* littermate male mice were selected as control mice. The animal experimental procedures were approved by the Hong Kong government, the Department of Health and the Animal Research Ethical Committee of the Chinese University of Hong Kong, and were consistent with the Guide for the Care and Use of Laboratory Animals published by the National Institutes of Health. The blood glucose level, insulin tolerance test, and oral glucose tolerance test were assessed as described previously.^16^ Urine samples were collected at 7, 11, and 15 weeks old of age by metabolic cages. The mice were sacrificed by CO_2_ anesthesia at 15 weeks old. The blood was collected from the posterior vena cava and immediately transferred to EP-tubes moistened with heparin sodium (25 U/mL in PBS) and centrifuged at 3000 rpm at 4 °C for 10 min. The supernatants were collected. All plasma and urine samples were stored under −80 °C until analysis.

### Determination of Urinary Creatinine Levels

Urinary creatinine levels were determined using a Creatinine Liquicolor kit (#0430-120, Stanbio Lab). In brief, 3 μL of urine samples and equal volumes of standards were added to a 96-well plate (in duplicates). A total of 135 μL of reagent 1 was added to each well and was incubated for 5 min at 37 °C. The absorbance (A1) was read immediately at 550 nm. Then, 45 μL of reagent 2 was added followed by incubation at 37 °C for 5 min. Then, the absorbance (A2) was read immediately at 550 nm. Blank samples were the same as urine samples but 3 μL of water was added instead. The urinary creatinine levels were calculated according to the manufacturer’s instructions.

### 3-Nitrophenylhydrazine Derivatization and Method Optimization

Method optimization was performed on an appropriate concentration of a mixed standard solution. Based on previous studies,^15,17,18^ the reaction temperature and reaction time were optimized. Briefly, 80 μL aliquots of standard solution were mixed with 40 μL of 3NPH-HCl solution [200 mM in 70% of methanol (methanol/H_2_O = 70/30, v/v)] and EDC-HCl solution [120 mM EDC (contained 6% pyridine) in 70% of methanol solution]. The reaction temperatures for optimization were set at −20 °C, 4 °C, 25 °C (room temperature), and 40 °C. The reaction times for optimization were set at 10, 30, 60, and 90 min.

### Derivatization of Plasma and Urine Samples

Serum samples (20 μL) were first deproteinized with four times volume of ice-cold methanol (containing appropriate concentrations of internal standards). The supernatants were collected, dried, and dissolved in 100 μL of 70% methanol solution. Then, 50 μL of 3NPH-HCl reaction solution and 50 μL of EDC-HCl reaction solution were added, mixed, and incubated at room temperature for 30 min. Urine samples were first diluted 20 times with 70% of methanol solution. A total of 40 μL of diluted urine samples was mixed with 40 μL of appropriate concentration of internal standard solution (in 70% of methanol). Then, 40 μL of 3NPH-HCl reaction solution and 40 μL of EDC-HCl reaction solution were added, mixed, and incubated at room temperature for 30 min.

### Method Validation

Stability, repeatability, and linearity were assessed. The stability of storage conditions under −80, −20, and 4 °C was evaluated. Additionally, inter- and intra-day stability was also determined at 8, 12, 24, 48, and 72 h at room temperature.

### Liquid Chromatography Coupled with Mass Spectrometry

Identification of 3-NPH-derivatized NAGlys was carried out using an Ultimate 3000 UHPLC (Dionex), combined with a Q Exactive Focus Orbitrap mass spectrometer (LC-QE-MS, Thermo Fisher Scientific, MA, USA). Separation was operated on a Phenomenex polar C18 column (1.6 μm, 2.1 × 150 mm). The mobile phase A was Milli-Q water with 0.01% FA, and the mobile phase B was ACN with 0.01% FA. The flow rate was 0.25 mL/min. The elution gradient was started with 30% of phase B, held for 1 min, gradually increased to 100% of phase B at 13 min, held for 2 min, then changed back to the initial composition of 30% B within 0.1 min, and held for 2.9 min.^16^ The column and sampler temperatures were kept at 40 and 25 °C, respectively. For the MS part, the resolution was 17,500. The capillary temperature was 320 °C. The spray voltage was assigned at 3.5 kV. The sheath gas was 30 psi, and the auxiliary gas was set at 10 psi. The collision energy was 25 eV. The detection was performed in parallel reaction monitoring (PRM) mode.

Targeted quantification of 3-NPH-derivatized NAGlys was performed on an UltiMate 3000 liquid chromatograph, coupled with a triple quadrupole mass spectrometer (LC-QqQ-MS) (Thermo Fisher Scientific, MA, USA). The parameters of liquid chromatography were the same as those on the LC-Orbitrap-MS. For the mass spectrometry part, the capillary temperature was 320 °C; the spray voltage was 2.5 kV, and the auxiliary gas heater temperature was 300 °C; the sheath gas and auxiliary gas flow rates were 40 and 10 psi, respectively. The quantification was employed in selected reaction monitoring mode. Transitions and collision energies of each 3-NPH derivatized NAGlys are listed in Table S1.

### Peak Extraction and Statistical Analysis

Raw data from LC-QqQ-MS were extracted by Thermo Xcalibur Processing Setup-QuanIdentification software. The background signal-to-noise ratio was assigned at 3. All the peaks were manually checked. The results from urine samples were normalized to urinary creatinine. Multivariate statistical analysis Partial Least-Squares Discriminant Analysis (PLS-DA) was performed using SIMCA-P (Umetrics, Umea, Sweden). Receiver operating characteristic curve (ROC) analysis was performed on MetaboAnalyst (https://www.metaboanalyst.ca/).^17^ Pathway analysis was carried out and referred to the KEGG pathway database (http://www.genome.jp/kegg/). Statistic significance was analyzed using Prism-GraphPad by Student’s *t*-test or analysis of variance (ANOVA) followed by Tukey’s multiple comparison test. Data are presented as mean ± se. A *P*-value less than 0.05 was regarded as statistically significant.

## Results and Discussion

### Derivatization Strategy of N-Acyl Glycines by 3-NPH

Functional-group-based targeted/non-targeted metabolomics is a promising strategy to capture low abundance metabolites, identify new metabolites, and expand the detection coverage. According to statistical analysis from metabolite databases (including HMDB, KEGG, YMDB, etc.), metabolites that contain carboxyl, carbonyl, and phosphoryl functional groups are more than 50% of the entire metabolome.^18^ The 3-NPH-based derivatization method is an effective strategy in targeting those compounds. Although previous studies have revealed that this method is effective in detecing NAGlys,^14,19^ the coverage of detectable NAGlys is still limited due to the lack of authentic standards.

The polarity of NAGlys varies from short-chain to long-chain NAGlys. Chromatographic separation of NAGlys before mass spectrometry detection, especially polar NAGlys, could enhance their response sensitivity (Figure 1b,c and Table S2). Derivatization of NAGlys (Figure 1a) by 3-NPH is effective in improving the retention of NAGlys on a reversed-phase column (Figure 1b,c), especially for glycine and short-chain NAGlys. In addition, the chromatography was also significantly improved, particularly for dicarboxylic acid-conjugated NAGlys (e.g., glutarylglycine). In this study, combined with the excellent quantification ability of high-resolution LC–MS on the reversed-phase column, the coverage of 3-NPH-derivatized NAGlys was greatly improved. More importantly, compared with other alkylation derivatization methods, such as *n*-butanol and methanol derivatization, 3-NPH derivatization can be performed in aqueous solution. When compared with the DmPA bromide derivatization, 3-NPH derivatization of NAGlys does not require high reaction temperatures and a quenching step. Therefore, this targeted 3-NPH-based strategy allowed comprehensive analysis of NAGlys with simple sample preparation procedures, which is promising in in-depth monitoring of the pathological changes of this species in relation with diseases, further facilitating the outcomes into areas of clinical applications.

### Optimization of Derivatization Conditions

To get a better detection performance, the 3-NPH derivatization conditions of NAGlys were optimized. As shown in Figure S1, almost all NAGlys displayed the highest MS response at room temperature (Figure S1a). In addition, we found that the yields of NAGlys reached maximum values at a reaction time of 30 min (with the RSDs of the yields of each NAGlys less than 10%) (Figure S1b). Finally, the derivatization conditions were optimized for reaction at room temperature for 30 min.

### Identification of N-Acyl Glycines in Urine and Plasma Samples

Derivatized NAGlys were first identified on a high-resolution LC-QE-MS platform by using PRM mode. Based on a previously published work,^8^ targeted PRM analysis was carried out based on characteristic fragmentations of 3-NPH-derivatized NAGlys (Table S1). Common fragments of 3-NPH-derivatized NAGlys were observed. The product ions *m/z* 122.0231, *m/z* 137.0339, and *m/z* 152.0450, which all came from the derivatization reagent 3-NPH, were observed. The specific and common fragment ions of NAGlys *m/z* 71.0251, *m/z* 166.0619, and *m/z* 209.0669 were observed and taken into consideration for NAGlys. Product ions owing to the loss of acyl chains were identified. For instance, *m/z* 86.0593, which belongs to the side chain of butyrylglycine, was found (Figure 1c,d). In addition to monocarboxylic NAGlys, dicarboxylic NAGlys was detected by 3-NPH derivatization (Figure 1e,f) as well. Each of the two carboxylic groups can be derivatized with 3-NPH. In addition to common fragments of NAGlys, a specific product ion *m/z* 319.1038, which is due to the loss of the 3-NPH derivatization reagent, was observed. Besides, *m/z* 262.0823, which resulted from the loss of the 3-NPH-derivatized glycine group, was identified. Owing to inadequate authentic standards, the identification of NAGlys is difficult. However, the accurate mass of parent and product ions improved the confidence of identification. Therefore, in this study, putative identification of 3-NPH-derivatized NAGlys was performed based on accurate MS, MS/MS spectra, and retention time. For the identification of NAGlys, at least two or more of the common fragments, *m/z* 152.0450, *m/z* 137.0339, or *m/z* 122.0231, due to the loss of 3-NPH or product ion of 3-NPH should be included. More importantly, the specific fragment ions of NAGlys, particularly *m/z* 209.0669, should be included in the identification of NAGlys. For those dicarboxylic NAGlys, the fragments with the loss of 3-NPH and glycine-conjugated 3-NPH must be included. Finally, a total of 41 NAGlys (including glycine) were identified from plasma and urine samples (Table S1).

### Method Validation and Sensitivity Evaluation of 3-NPH Derivatization of NAGlys

Based on the identification of NAGlys by high-resolution LC-QE-MS, quantification was carried out on an LC-QqQ-MS. Collision energies and transitions of each NAGlys were optimized and are listed in Table S1. Stability assessment revealed that compared with storage conditions at 4 °C, keeping the derivatized samples under −20 or −80 °C improved the stability (Figure S1c). Samples were stable within 48 h at room temperature, as revealed by inter- and intra-day stability evaluation (Figure S1d). The repeatability was evaluated in low, medium, and high concentrations. The results indicated that the 3-NPH derivatization method achieved good repeatability for the detection of NAGlys (Table S2). The linearity study revealed that this method displayed good quantification ability (Table S2). More importantly, the sensitivity was improved enormously, especially for short- to medium-chain NAGlys (improved more than 50 times) (Table S2). Besides, it is worth mentioning that owing to poor retention capacity, it is hard to quantify glycine with the other NAGlys without derivatization simultaneously in one injection. However, it can be detected together with the other NAGlys simultaneously within one injection after 3-NPH derivatization. The current method could substantially enlarge the detection scope.

### Targeted Profiling Analysis of N-Acyl Glycines in Plasma Samples of *db/db* Mice

**Figure 2:**
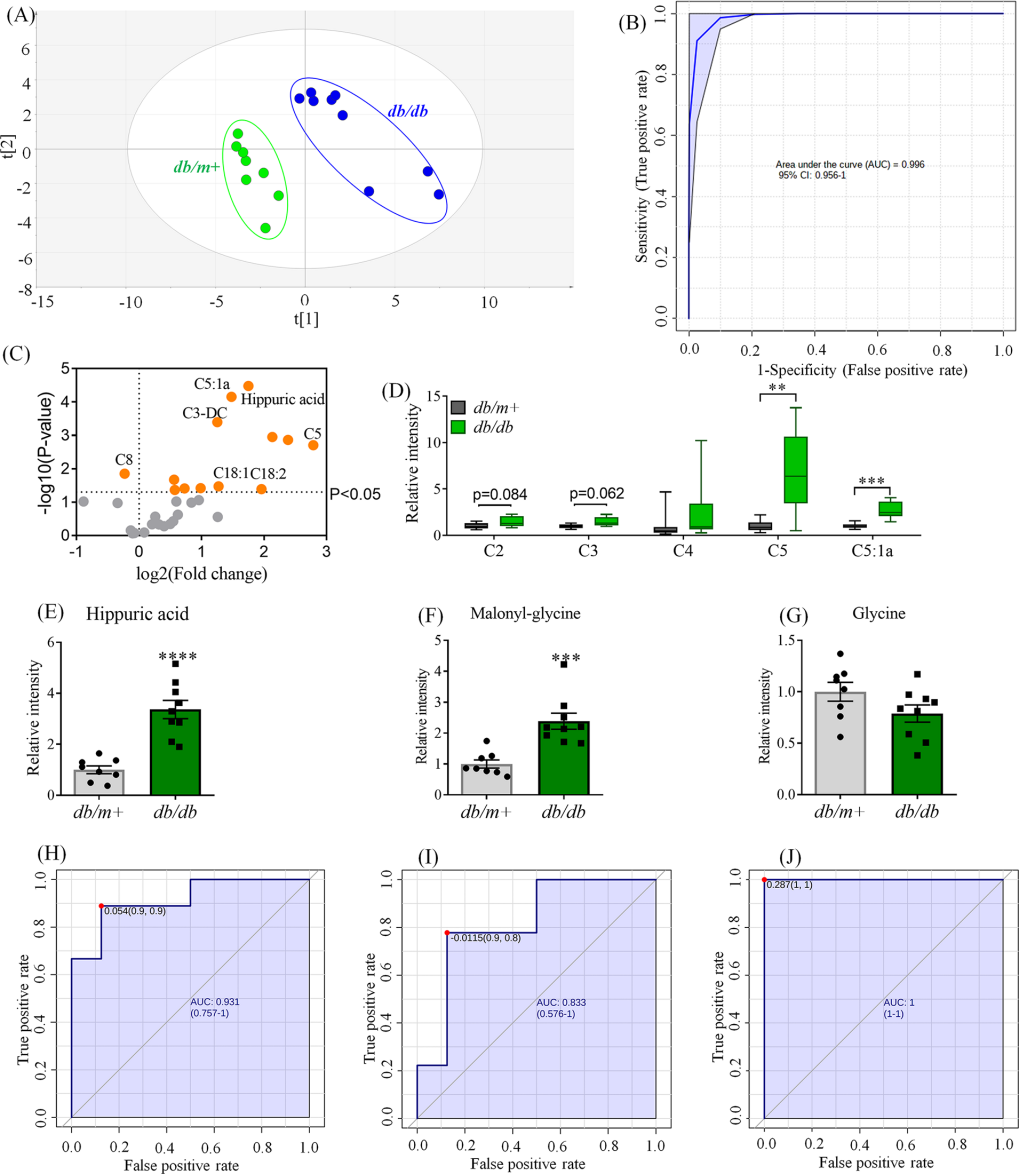
NAGlys in plasma of *db/db* mice. (A) PLS-DA score plot of NAGlys of *db/m+* (Green dots) and *db/db* (blue dots) mice. (B) Receiver operating characteristic curve analysis (ROC) of NAGlys in discriminating *db/m+* and *db/db* mice. The ROC analysis was operated on MetaboAnalyst (www.metaboanalyst.ca). AUC, area under the curve. (C) Volcano plot of NAGlys in a plasma sample. C5, valeryl/isovaleryl/2-methylbutylglycine. C5:1a, tiglyglycine. C3-DC, malonylglycine. C18:1, oleoylglycine. C18:2, linoleoylglycine. (D) Short-chain NAGlys in plasma. C2, acetylglycine. C3, propionylglycine. C4, butyl/isobutylglycine. Levels of (E) hippuric acid, (F) malonylglycine, and (G) glycine in plasma. ROC analysis of (H) hippuric acid, (I) malonylglycine, and (J) glycine in plasma. **, *p* < 0.01; ***, *p* < 0.001; ****, *p* < 0.0001, *db/db* vs *db/m+*.

*db/db* mice have long been applied in the study of T2DM.^20^ In this study, targeted analysis of NAGlys in plasma of *db/db* mice was carried out. The PLS-DA score plot shows that *db/db* diabetic mice are clearly separated from *db/m+* control mice (Figure 2a). The ROC analysis (the value of area under the curve (AUC) = 0.996) (Figure 2b) indicated that the contents of NAGlys were significantly different in mice with/without diabetes. The volcano plot revealed that various kinds of NAGlys were disturbed in the diabetic mice group compared to the *db/m+* control group (Figure 2c). Almost all short-chain NAGlys were substantially increased (Figure 2d), which may indicate the dysfunction of both amino acid and fatty acid metabolism in response to T2DM. Additionally, the levels of several long-chain NAGlys were significantly accumulated in the plasma (Figure 2c). Consequently, the medium-chain NAGlys such as octanoylglycine (C8) decreased, which may be due to the dysfunction of fatty acid beta-oxidation (FAO).^5^ Previous studies indicated that accumulation of fatty acids contributed to insulin resistance.^21,22^ Excretion of fatty acids in the form of N-acyl glycine conjugates through urine is an effective way to eliminate excess free fatty acids.^23^ Therefore, the alterations of short- to long-chain NAGlys in plasma may indicate the dysfunction of FAO in response to T2DM.

Hippuric acid is the end product of phenylalanine and tyrosine produced by the gut microbiota.^24^ In this study, the levels of hippuric acid together with hydroxyl-conjugated hippuric acid were significantly increased in the plasma of *db/db* mice (Figure 2e and Figure S3a). Moreover, we also found high distinguishing abilities between *db/m+* and *db/db* mice (Figure 2h and Figure S3b). The accumulation of hippuric acid has been reported positively related to the estimated glomerular filtration rate (eGFR)^25^ and other risk factors of kidney failure and dysfunction.^26,27^ In addition, hippuric acid has also been reported to be related to cardiovascular diseases via promoting endothelial dysfunction^28,29^ and contribute to hepatic damage in hepatic disease.^30^ Therefore, hippuric acid may be considered as a potential biomarker for diabetes and diabetic complications. Additionally, in the current study, malonylglycine was detected in the plasma of *db/db* mice for the first time. It was considerably increased in the *db/db* group (Figure 2f) and exhibited high separation ability between *db/m+* and *db/db* mice (Figure 2i). Accordingly, observation of the changes of malonylglycine may be applicable in the diagnosis of T2DM.

Consequently, owing to the accumulation of glycine conjugates, the level of free glycine was slightly decreased in the plasma from diabetic mice (Figure 2g), but the AUC value was capable to reach 1 (Figure 2j), indicating a high differentiating ability between *db/m+* and *db/db* mice. The finding was consistent with a previous study that the plasma-free glycine level is negatively correlated to diabetes^31^ and positively correlated to insulin sensitivity.^32^ All the above results of free glycine and glycine conjugates in plasma revealed that metabolites in the glycine metabolic pathways were substantially affected under diabetic conditions. Moreover, observing those species of metabolites may lead to discovery of novel biomarkers for T2DM.

### Targeted Profiling Analysis of N-Acyl Glycines in Urine Samples of *db/db* Mice

**Figure 3:**
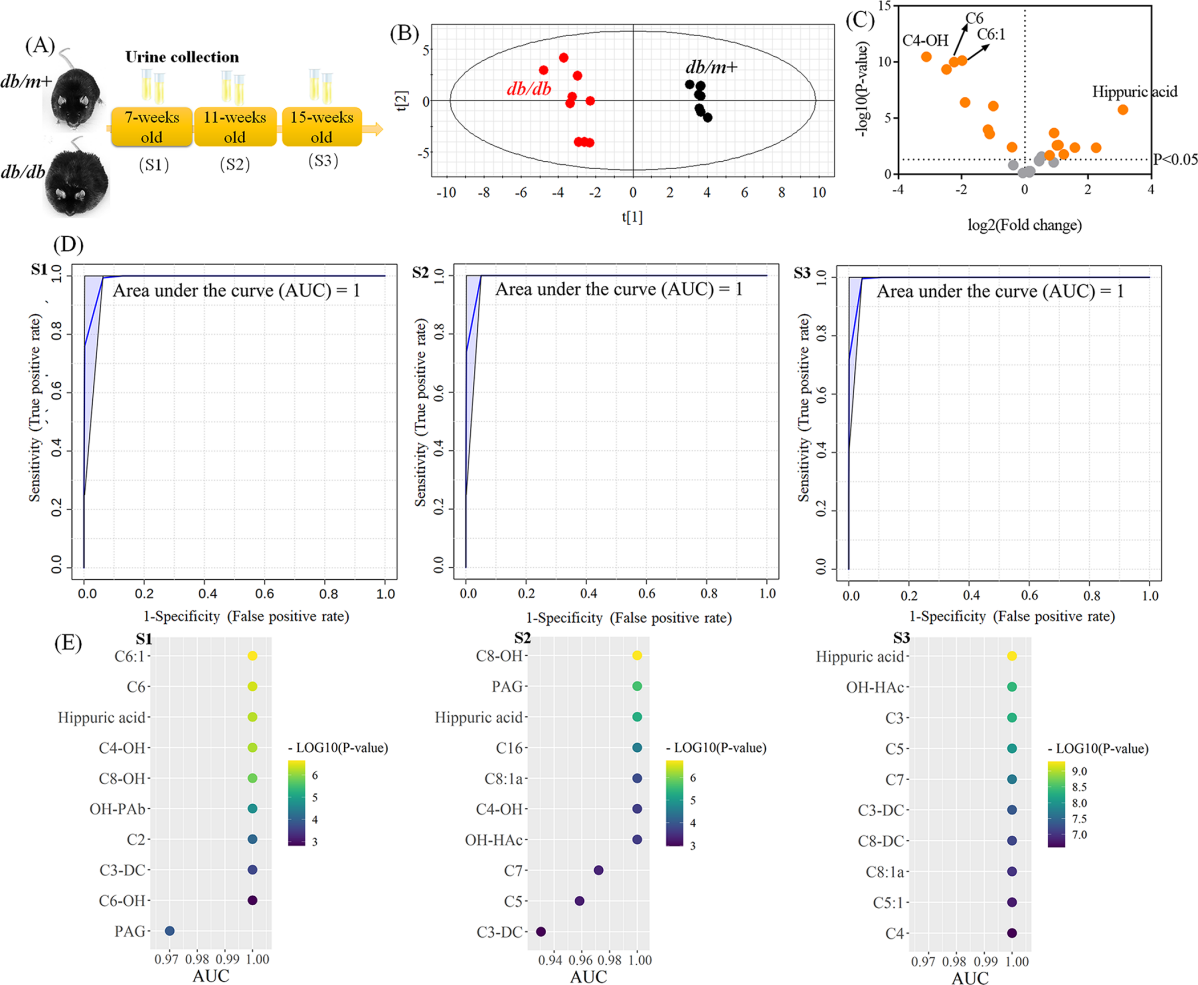
Targeted analysis of NAGlys in urine samples of *db/db* mice. (A) Schematic diagram of animal experiments. S1, the first stage, 7 weeks old. S2, the second stage, 11 weeks old. S3, the third stage, 15 weeks old. (B) PLS-DA score plot of NAGlys in urine samples from the first stage (S1). (C) Volcano plot of NAGlys in urine samples from S1. (D) ROC analysis of all detected NAGlys in urine from the three stages. AUC, area under the curve. The ROC analysis was performed on MetaboAnalyst (https://www.metaboanalyst.ca/). (E) Urinary NAGlys (top 10) contribute to the discrimination of *db/db* and *db/m+* mice based on ROC analysis.

T2DM is a typical metabolism disease. Accumulation of fatty acids, amino acids, and other metabolic intermediates contributing to the progression of T2DM was frequently observed.^33^ Since the collection of urine samples was convenient and non-invasive, monitoring the metabolic end products in urine was practical and efficient in clinical application. A wealth of metabolic studies were carried out to investigate the alteration of metabolites in urine samples^34,35^ under diabetic conditions. Among the altered metabolites, urinary NAGlys are the primary class of metabolites in the detoxification system of the human body. Therefore, a comprehensive observation of urinary NAGlys is valuable in early diagnosis and progression monitoring of T2DM. In this study, targeted profiling analysis of NAGlys in urine samples was performed at three stages (S1, 7 weeks old; S2, 11 weeks old; S3, 15 weeks old, Figure 3a). The NAGlys content in urine displayed clear separations between all stages (Figure 3b and Figure S4). Most of them were altered in the urine even at the early stage (Figure 3c). Additionally, excellent differentiation ability (AUC = 1) was obtained between *db/m+* and *db/db* mice at all the three stages (Figure 3d). Several NAGlys, such as C3-DC, hippuric acid, and short-chain fatty acyl glycines all extensively contributed to the discrimination of the *db/db* mice and *db/m+* mice in urine samples (Figure 3e).

**Figure 4:**
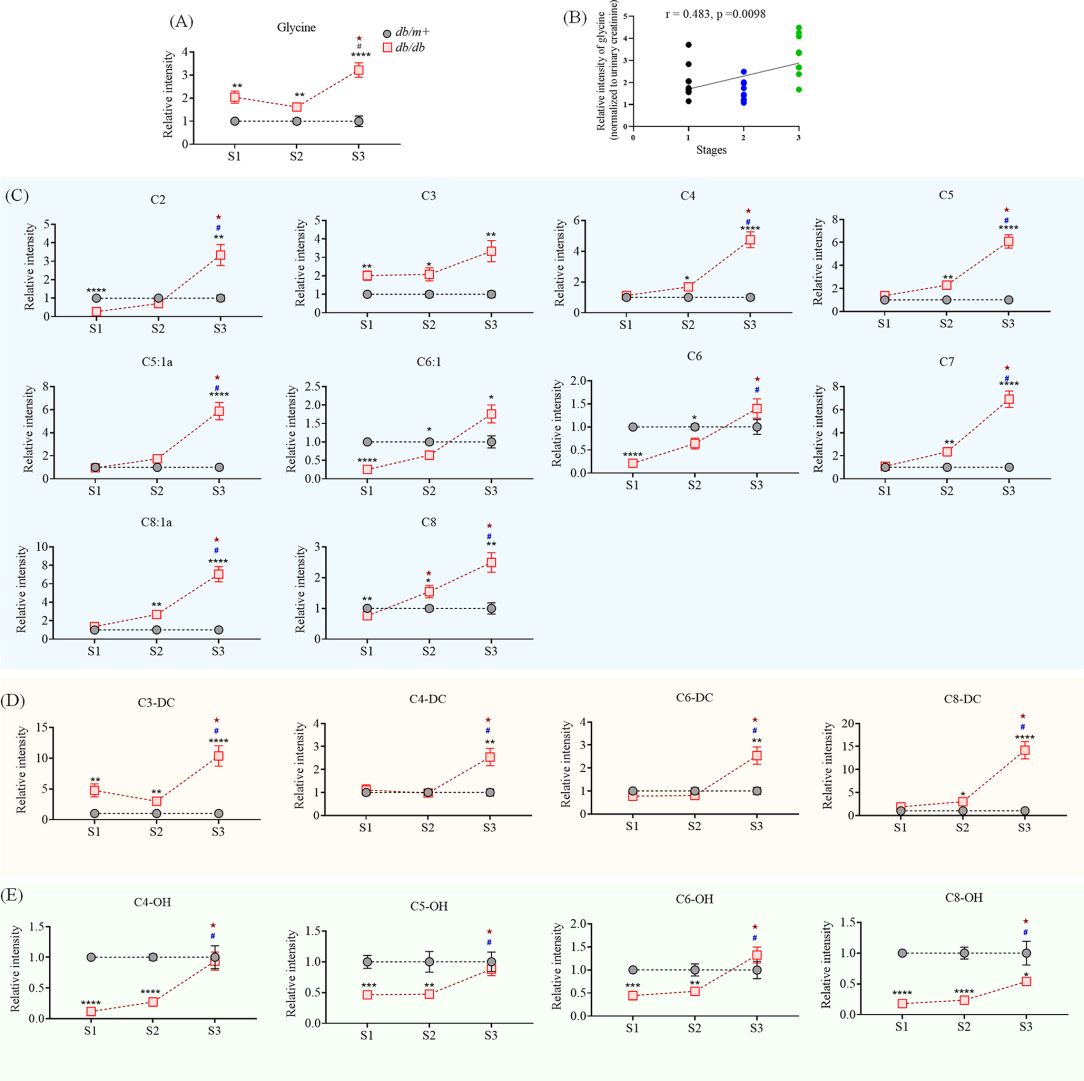
Changes of NAGlys at three stages in urine samples. (A) Changes of glycine. (B) Correlation analysis of glycine in the progression of T2DM in urine samples. (C) Changes of short- and medium-chain NAGlys at three stages. C2, acetylglycine. C3, propionylglycine. C4, butyl/isobutylglycine. C5, valeryl/isovaleryl/2-methylbutylglycine. C5:1a, tiglyglycine. C6:1, hexenoylglycine. C6, hexanoylglycine. C7, heptanoylglycine. C8:1a, octenoylglycine. C8, octanoylglycine. (D) Changes of dicarboxyl-conjugated NAGlys at three stages. C3-DC, malonylglycine. C4-DC, succinyl/methylmalonylglycine. C6-DC, adipoylglycine. C8-DC, suberoylglycine. (E) Changes of hydroxyl-conjugated NAGlys at three stages. C4-OH, 3-hydroxyiso/butyrylglycine. C5-OH, 3-hydroxyvaleryl/isovalery/2-methylbutyrylglycine. C6-OH, hydroxyhexanoylglycine (hydroxyl position unknown). S1, the first stage, 7-weeks old. S2, the second stage, 11-weeks old. S3, the third stage, 15-weeks old. *, *p* < 0.05; **, *p* < 0.01; ***, *p* < 0.001; ****, *p* < 0.0001, *db/db* vs *db/m+*. solid star, p < 0.05, *db/db* sample, S3/S2 vs S1; #, *p* < 0.05, *db/db* sample, S3 vs S2.

When investigating the changes of individual NAGlys in response to the progression of T2DM in the urine, we found that the excretion of glycine in the urine was gradually increased, and significance was found between S3 and S1/S2 (Figure 4a), which were consistent with our findings in the plasma(Figure 2g). The results implied that the loss of the detoxification material glycine was positively corelated to the progress of diabetes (Figure 4b). Additionally, the changes of short- and medium-chain NAGlys were also examined. The initial level of acetylglycine was notably reduced in S1 in the urine of *db/db* mice compared with *db/m+* lean control mice. Then, the excretion of acetylglycine was gradually elevated at early stages and enormously increased at S3 (Figure 4c). A similar phenomenon was observed in the changes of the other short- to medium-chain NAGlys [propionylglycine (C3), isobutyryl/butyrylglycine (C4), isovaleryl/valeryl/2-methylbutyrylglycine (C5), tiglyglycine (C5:1a), hexanoylglycine (C6), mono-unsaturated hexanoylglycine (C6:1), heptanoylglycine (C7), octenoylglycine (C8:1a), and octanoylglycine (C8)]. This may owe to renal dysfunction of reabsorption in the progression of diabetes.

Dicarboxylic acyl-conjugated NAGlys were also detected in the urine. The level of malonylglycine (C3-DC) was substantially raised at the early stage of diabetes and then dramatically elevated at 15 weeks of age. Succinyl/methylmalonylglycine (C4-DC), adipoylglycine (C6-DC), and suberoylglycine (C8-DC) were not notably altered at the early stage of diabetes. However, they were dramatically elevated at 15 weeks of age and exhibited high differentiating ability between control and diabetic mice (Figure 3E and Figure 4). The results indicated that C3-DC might be employed as a diagnosis marker for the early stage of diabetes, while C4-DC, C6-DC, and C8-DC may be used as diagnostic markers at the late stage.

Interestingly, several hydroxyl-conjugated NAGlys were found substantially reduced at the early and middle stages in the urine of *db/db* mice (Figure 4d). Excellent discrimination abilities were found (AUC = 1) at the early stage (Figure 3E and Figure 4). However, their levels were significantly increased at the late stage when compared with the early and middle stages. The dramatic reduction of hydroxyl-conjugated NAGlys at the early stage may resulted from increased circulating levels of hydroxyl-conjugated short-chain fatty acids, such as BCAA metabolite 3-hydroxyisobutyric acid, which is toxic to the body by promoting insulin resistance.^36^ Therefore, observation of hydroxyl-conjugated NAGlys probably could facilitate the early diagnosis of diabetes.

Besides, the levels of several phenyl derivative-conjugated NAGlys were also significantly increased at the early stage of T2DM. The level of hippuric acid was increased from the early stage of diabetes in the urine (Figure S5). The levels of hydroxyl-conjugated hippuric acid (OH-HA), hydroxylphenylacetylglycine (OH-PA), phenylpropionylglycine (PPG), and phenylacetylglycine (PAG) were all gradually elevated with the progression of diabetes, which was contradictory with previous reports that phenol derivative-conjugated glycines such as hippuric acid and OH-HU were decreased in urine samples of *db/db* mice.^37^ The reason might be due to different normalization methods. The previous studies were presented as absolute metabolite concentrations in the urine, and the levels of hippuric acid in the current studies were presented as relative levels normalized to urinary creatinine. When we normalized their levels to urinary volume (urine collection for 24 h), we found that most of the levels of phenyl derivative-conjugated glycines were notably decreased in the urine of diabetic mice (Figure S6), especially at S1 and S2. The results were consistent with the other studies that the levels of hydroxyl-conjugated hippuric acid were decreased in the urine of pre-diabetic individuals.^38^ Nevertheless, dramatic increases were also observed at S3 compared with S1, indicating the severe renal failure of the reabsorption ability at the end-stage.

## Conclusions

In summary, a new derivatization method for comprehensive detection of NAGlys by the LC–MS platform was established. Compared with other derivatization methods, the current established method for detecting NAGlys is easier to operate, and satisfactory sensitivity and linearity were achieved. The tested stability and repeatability can satisfy large-scale sample analysis. Comprehensive analysis of NAGlys in plasma and urine samples revealed enormous metabolic changes between *db/m+* lean control and *db/db* diabetic mice. Most of the detected NAGlys were changed even at the early stage of T2DM both in plasma and urine samples. Several altered NAGlys, such as malonylglycine and succinyl/methylmalonylglycine, were first observed in the plasma and urine of *db/db* mice. Additionally, most altered NAGlys exhibited good capabilities in differentiating control and diabetic mice. However, the current method has limitations in separating isomers. Several isomers, such as butyrylglycine and isobutyrylglycine, cannot be separated well from each other. More efforts will be taken to solve this problem in the future.

## Supporting Information Available

Details of LC–MS detection information of N-acyl glycines, derivatization optimization of 3-NPH method, method validation, and additional data of N-acyl glycines in plasma and urine samples (Tables S1–S3 and Figures S1–S6) (PDF)
