# Supplementary material for: Derivatization of N-Acyl Glycines by 3-Nitrophenylhydrazine for Targeted Metabolomics Analysis and Their Application to the Study of Diabetes Progression in Mice
Source: Anal Chem. 2023 Jan 19;95(4):2183–91. doi: 10.1021/acs.analchem.2c02507 (PMC9893217; doi:10.1021/acs.analchem.2c02507)
Supplement: Supplementary file 1 — ac2c02507_si_001.pdf [file ac2c02507_si_001.pdf]

Supporting information

Derivatization of N-acyl glycines by 3-Nitrophenylhydrazine for targeted metabolomics analysis and its application to study diabetes progression in mice

Li Xiang[a#], Yi Ru[a#], Jingchun Shi[a], Li Wang[b], Hongzhi Zhao[c], Yu Huang[b*], Zongwei Cai[a*]

a. State Key Laboratory of Environmental and Biological Analysis, Department of Chemistry, Hong Kong Baptist University, Hong Kong 999077, China
b. Department of Biomedical Sciences, City University of Hong Kong, Hong Kong 999077, China
c. Ministry of Education Key Laboratory of Pollution Processes and Environmental Criteria, College of Environmental Science and Engineering, Nankai University, Tianjin 300350, China

* Correspondence to: Yu Huang, PhD, Email yu.huang@cityu.edu.hk; Zongwei Cai, PhD, Email zwcai@hkbu.edu.hk

# Li Xiang and Yi Ru contributed equally

Table of Contents

Table S1 Detailed information of 3-NPH derivatized N-acyl glycines........................3

Table S2 Linearity and LOD of the derivatization method on N-acyl glycines...........7

Table S3 Repeatability of the 3-NPH derivatization method on N-acyl glycines........8

Figure S1 Method optimization of 3-NPH derivatization of N-acyl glycines. 9

Figure S2 Pharmacological parameters of *db/m+* and *db/db* mice at the age of 15-weeks old..9

Figure S3 Changes of hydroxyl-hippuric acid in plasma of *db/m+* and *db/db* mice. .10

Figure S4 Multivariate statistical analysis based on detected N-acyl glycines in urine samples. 11

Figure S5 Changes of phenyl derivative-conjugated glycines in urine samples.........12

Figure S6 Changes of phenyl derivative-conjugated glycines in urine......................13

Table S1 Detailed information of 3-NPH derivatized N-acyl glycines.

NO.	Compounds	Abbreviations	*m/z* (3-NPH conjugation, [H⁻])	R.T.	Parent/Product ions (*m/z*)	CE (eV)	MS/MS fragments (*m/z*)
**1**	Glycine[std]	C0	209.0680	2.1	209.1/137.1	20	166.0607, 152.0450, 137.0340, 71.0235
**2**	Dimethylglycine	2Me-C0	237.0993	2.2	237.1/137.1	15	209.0669, 152.0450, 137.0340, 71.0234
**3**	Acetylglycine[std]	C2	251.0786	2.6	251.1/209.1	15	251.0782, 209.0670, 166.0616, 152.449, 137.0339, 122.0230,71.0238
**4**	3-Hydroxyiso/butyrylglycine	C4-OH	295.1048	2.6	295.1/209.1	20	251.0777, 209.0667, 152.0499, 137.034, 58.0277
**5**	Propionylglycine[std]	C3	265.0942	3.1	265.1/209.1	20	265.0538, 209.0667, 152.0450, 137.0340, 122.0232, 107.0358, 71.0238
**6**	3-Hydroxyvaleryl/isovalery/2-methylbutyrylglycine	C5-OH	309.1204	3.6	309.1/209.1	20	309.1200, 265.0942, 237.0980, 209.0684, 152.0451, 137.0340, 122.0233, 88.0387, 72.0443
**7**	Hydroxyhippuric acid (hydroxyl position unknown)	OH-HAa	329.0891	3.9	329.1/209.1	20	329.0884, 209.0668, 152.0446, 137.0338, 122.0232, 71.0234
**8**	Isobutyryl/butyrylglycine[std]	C4	279.1099	4.0	274.1/209.1	20	279.1092, 209.0669, 152.0450, 137.0339, 86.0593, 71.0239
**9**	Hydroxyphenylacetylglycine (hydroxyl position unknown)	OH-PAa	343.1048	4.0	343.1/209.1	20	343.1045, 237.0629, 209.0672, 152.0450, 137.0340, 122.0233, 71.0239
**10**	Hydroxyhippuric acid (hydroxyl position unknown)	OH-HAb	329.0891	4.2	329.1/209.1	20	329.0883, 209.0673, 152.0446, 137.0339, 136.0387, 122.0233, 118.0281, 71.0251
**11**	Hydroxyhexanoylglycine (hydroxyl position unknown)	C6-OH	323.1361	4.3	323.1/209.1	20	323.1355, 305.1261, 251.0781, 209.0669, 166.0623, 152.0449, 137.0339, 122.0231, 71.0240

**12**	Tiglyglycine	C5:1a	291.1099	4.7	291.1/209.1	20	291.1094, 209.0669, 152.0450, 137.0340, 122.0231, 98.0595, 71.0235
**13**	3-Methylcrotonylglycine	C5:1b	291.1099	4.8	291.1/209.1	20	291.1095, 209.0669, 152.0449, 137.0340, 122.023298.0592, 71.0240
**14**	2-Methylbutyryl/isovaleryl[std]/valerylglycine	C5	293.1255	4.9	293.1/209.1	20	293.1254, 209.0669, 152.0450, 137.0340, 122.0232, 100.0751,71.0234
**15**	Hydroxyhippuric acid (hydroxyl position unknown)	OH-HAc	329.0891	4.9	329.1/209.1	20	329.0876, 209.0673, 152.0451, 122.0235, 71.0239
**16**	Malonylglycine	C3-DC	430.1118	4.9	430.1/209.1	25	430.1113, 277.0575, 235.0466, 220.0356, 209.0669, 152.0451, 137.0340, 122.0232, 71.0239
**17**	Succinyl/methylmalonylglycine	C4-DC	444.1273	5.0	444.1/209.1	25	444.1264, 291.0731, 234.0513, 209.0669, 192.0403, 152.0454, 137.0340, 122.0232, 71.0240
**18**	Glutarylglycine[std]	C5-DC	458.1434	5.1	458.1/209.1	25	458.1434, 305.0894, 248.0678, 209.0674, 166.0907, 152.0452, 137.0343, 122.0235, 71.0232
**19**	Adipoylglycine[std]	C6-DC	472.1586	5.2	472.1/209.1	30	472.1574, 319.1040, 262.0825, 209.0669, 178.0245, 152.0450, 150.0186, 137.0340, 122.0232, 71.0238
**20**	Hydroxyoctanoylglycine (hydroxyl position unknown)	C8-OH	351.1674	5.3	351.1/209.1	20	351.1666, 209.0654, 152.0450, 137.0340, 122.023, 71.0231
**21**	Hippuric acid[std]	HA	313.0942	5.3	313.1/137.1	20	313.0942, 175.0698, 152.0449, 137.0341, 122.0235, 120.0439
**22**	Hydroxyphenylacetylglycine (hydroxyl position unknown)	OH-PAb	343.1048	5.5	343.1/209.1	20	343.1041, 209.0655, 152.0449, 150.0544, 137.0339, 122.0232, 71.0233

**23**	Methylhippuric acid (methyl position unknown)	Me-HA	327.1099	5.6	327.1/209.1	20	327.1092, 209.0668, 152.0450, 137.0339, 122.0232, 71.0237
**24**	Phenylacetylglycine (PAG)	PAG	327.1099	5.7	327.1/209.1	20	327.1092, 209.0668, 152.0446, 137.0339, 71.0223
**25**	Hexenoylglycine (double bond position unknown)	C6:1	305.1255	5.8	305.1/209.1	20	305.1254, 261.0986, 209.0686, 152.0451, 137.0340, 112.0750
**26**	Suberoylglycine^{std}	C8-DC	500.1899	5.9	500.1/209.1	30	500.1889, 347.1353, 290.1143, 209.0669, 178.0249, 152.0449, 150.0187, 137.0339, 122.0223, 71.0239
**27**	Hexanoylglycine^{std}	C6	307.1412	6.0	307.1/209.1	20	307.1407, 209.0668, 152.0451, 137.0340, 114.0906, 71.0240
**28**	Phenylpropionylglycine	PPG	341.1255	6.1	341.1/209.1	20	341.1251, 209.0685, 152.0465, 137.0340, 122.0233
**29**	Octenoylglycine	C8:1a	333.1566	7.0	333.2/209.1	20	333.1566, 209.0667, 152.0449, 140.1065, 137.0339, 122.0234, 71.0238
**30**	Heptanoylglycine	C7	321.1568	6.7	321.2/209.1	20	321.1560, 209.0688, 152.0460, 137.0348, 128.1077
**31**	Phenylbutyrylglycine	PBG	355.1412	7.3	355.1/209.1	20	355.1409, 209.0671, 162.0926, 152.0453, 137.0340, 122.0232, 71.0240
**32**	Octenoylglycine	C8:1b	333.1566	7.4	333.2/209.1	20	333.1566, 209.0680, 152.0450, 140.1066, 122.0235
**33**	Octanoylglycine^{std}	C8	335.1725	7.5	335.2/209.1	23	335.1725, 209.0668, 152.0450, 142.1223, 137.0340, 122.0233, 71.0240
**34**	Myristoylglycine	C14	419.2664	11.3	419.3/209.1	25	419.2647, 283.2635, 209.0685, 152.0455, 137.0341, 122.0235

**35**	Palmitoleoylglycine	C16:1	445.2820	11.6	445.3/209.1	25	445.2805, 209.0681, 152.0458, 137.0340, 71.0238
**36**	Docosahexaenoylglycine	C22:6	519.2977	11.8	519.3/209.1	25	519.2960, 263.0781, 209.0669, 152.0456, 137.0339, 122.0231, 71.0237
**37**	Arachidonoylglycine	C20:4	495.2977	11.8	495.3/209.1	25	495.2977, 223.0835, 209.0672, 152.0455, 137.0339, 122.0232, 71.0239
**38**	Linoleoylglycine	C18:2	471.2977	11.9	471.3/209.1	25	471.2959, 209.0669, 152.0458, 137.0338, 122.0231, 71.0237
**39**	Palmitoylglycine[std]	C16	447.2977	12.4	447.3/209.1	25	447.2970, 279.2321, 254.2482, 209.0667, 152.0449, 137.0338, 122.0234, 107.0356, 71.0238
**40**	Oleoylglycine	C18:1	473.3133	12.6	473.3/209.1	25	473.3143, 280.2644, 209.0669, 178.0259, 152.0447, 137.0337, 122.0233, 107.0356, 71.0235
**41**	Stearoylglycine	C18	475.3290	13.3	475.3/209.1	25	475.3272, 322.2738, 282.2793, 209.0665, 166.0618, 152.0446, 137.0337, 122.0233, 107.0356, 84.0072, 71.0237

Note: R.T., retention time. *m/z*, mass to charge ratio. [std] represents validated by authentic references.

Table S2 Linearity and LOD of the derivatization method on N-acyl glycines.

N-Acyl glycines	Urine matrix			Plasma matrix			LOD (ng/mL)	
	Slope	R^2	Range (μg/mL)	Slope	R^2	Range (μg/mL)	3-NPH derivatized	No derivatization
Glycine	0.0651	0.9846	0.021-5.0	0.0174	0.9972	0.2-5.0	11.1	50
C2	0.4839	0.9919	0.008-5.0	0.0606	0.9919	0.02-5.0	0.085	5
C3	0.4829	0.9961	0.002-5.0	0.1291	0.9894	0.007-5.0	0.028	5
C4	1.0028	0.9984	0.0008-5.0	0.6818	0.9994	0.002-5.0	0.028	2
C5	0.8045	0.9991	0.0008-5.0	0.5952	0.9999	0.008-5.0	0.085	5
C6	0.2886	0.9976	0.0008-5.0	0.2097	0.9992	0.008-5.0	0.028	2
C8	0.5511	0.9998	0.0008-5.0	0.3611	0.9998	0.007-5.0	0.028	2
C16	0.3163	0.9993	0.02-5	0.223	0.9931	0.007-5.0	0.2	1
C5-DC	1.7996	0.9998	0.007-5.0	1.2807	0.9997	0.002-5.0	0.028	5
C6-DC	0.8587	0.9991	0.0008-5.0	0.6196	0.9984	0.08-5.0	0.085	2
C8-DC	0.2988	0.9995	0.002-5.0	0.2106	0.9995	0.0008-5.0	0.028	1
Hippuric acid	0.5104	0.9958	0.007-5.0	0.3322	0.9998	0.06-5.0	0.254	5

Note: The calibration curve of glycine in urine matrix was present as logarithmic trend curve. Other curves in either urine or plasma matrix were presented as linear curve. A matrix blank sample was prepared. All the response rations for the generation of curves was deducted of the response ratios already existed in the matrix blank sample. LOD, limit of detection. LOD was performed by using authentic standards, and was determined as the concentration when the corresponded value of signal/noise ratio equal or more than 3. C2, acetylglycine. C3, propionylglycine. C4, butyrylglycine. C5, valerylglycine. C6, hexanoylglycine. C8, octanoylglycine. C16, palmitoylglycine. C5-DC, glutarylglycine. C6-DC, adipoylglycine. C8-DC, suberoylglycine. LOD, limit of detection.

Table S3 Repeatability of the 3-NPH derivatization method on N-acyl glycines.

N-acyl glycines	High concentration (500 ng/mL, RSD %)	Medium concentration (50 ng/mL, RSD %)	Low concentration (5 ng/mL, RSD %)
C2	3.46	2.23	7.19
C3	4.11	2.34	2.55
C4	6.00	2.14	3.48
C5	2.42	2.52	1.10
C6	2.71	2.73	4.14
C8	4.00	3.45	4.24
C16	1.77	6.21	2.27
C5-DC	1.68	7.08	0.66
C6-DC	2.74	3.49	2.85
C8-DC	1.91	5.81	6.89
Hippuric acid	3.08	3.84	6.95

Note: Repeatability was calculated as RSD % of repeat injection of the same sample at low (5 ng/mL), medium (50 ng/mL) and high concentrations (500 ng/mL), respectively. C2, acetylglycine. C3, propionylglycine. C4, butyrylglycine. C5, valerylglycine. C6, hexanoylglycine. C8, octanoylglycine. C16, palmitoylglycine. C5-DC, glutarylglycine. C6-DC, adipoylglycine. C8-DC, suberoylglycine.

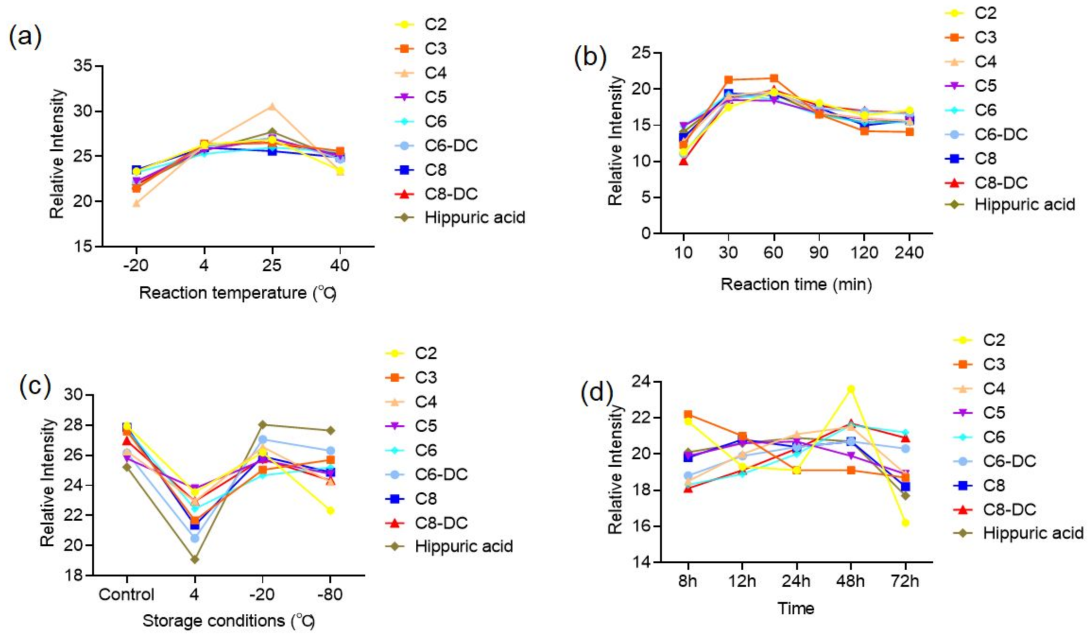

Figure S1 Method optimization of 3-NPH derivatization of N-acyl glycines.
(a) Optimization of reaction temperature. n=3. (b) Optimization of reaction time. n=5. (c) Evaluation of long-term storage conditions. Data was acquired after 7 days of 3-NPH derivatization under different storage conditions. Control group was acquired from freshly prepared samples. n=5. (d) Evaluation of intro- and inter-day stability under room temperature °C. n=5. C2, acetylglycine. C3, propionylglycine. C4, butyrylglycine. C5, isovalerylglycine. C6, hexanoylglycine. C8, octanoylglycine. C6-DC, adipoylglycine. C8-DC, suberoylglycine. The data was normalized to the sum of tested conditions, and was presented as mean of normalized value.

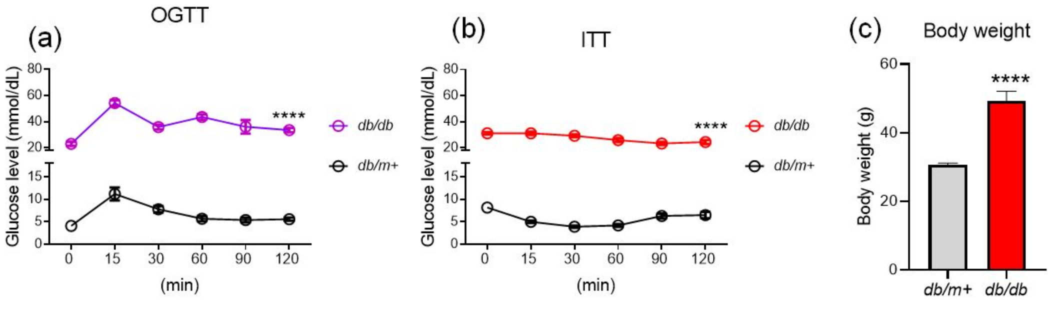

Figure S2 Pharmacological parameters of *db/m+* and *db/db* mice at the age of 15-weeks old.
(a) Oral glucose tolerance test (OGTT). (b) Insulin tolerance test (ITT). (c) Body weight. n=8-9. ****, $p < 0.0001$, *db/db* vs *db/m+*. Data were analyzed by student t-test, and presented as mean ± se.

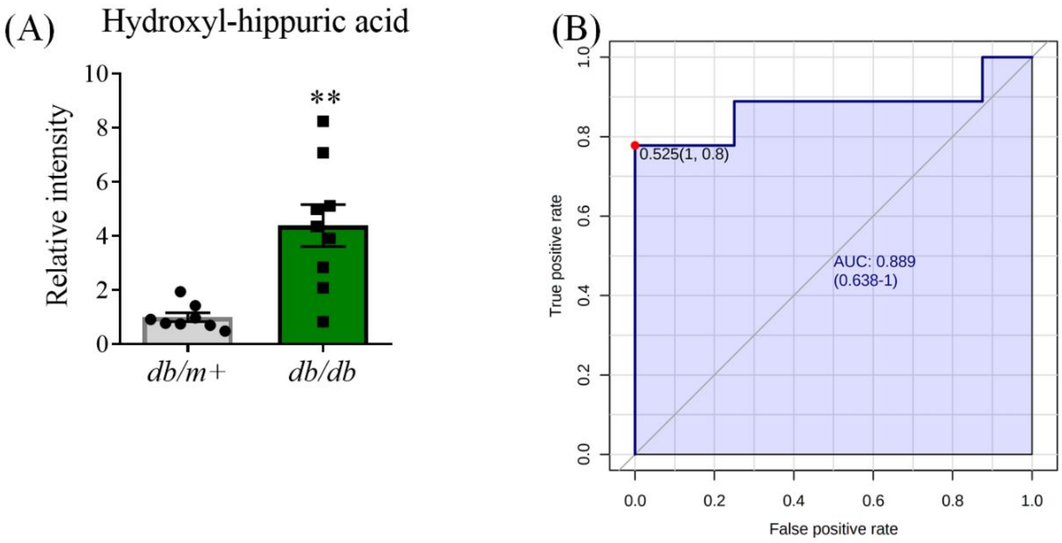

Figure S3 Changes of hydroxyl-hippuric acid in plasma of *db/m+* and *db/db* mice. (A) Statistic analysis of hydroxyl conjugated hippuric acid in plasma. Data were analyzed by student t-test, and presented as mean ± se. (B) ROC analysis of hydroxyl conjugated hippuric acid. AUC, area under curve. **, $p < 0.01$, *db/db* vs *db/m+*. Data were analyzed by student t-test, and presented as mean ± se.

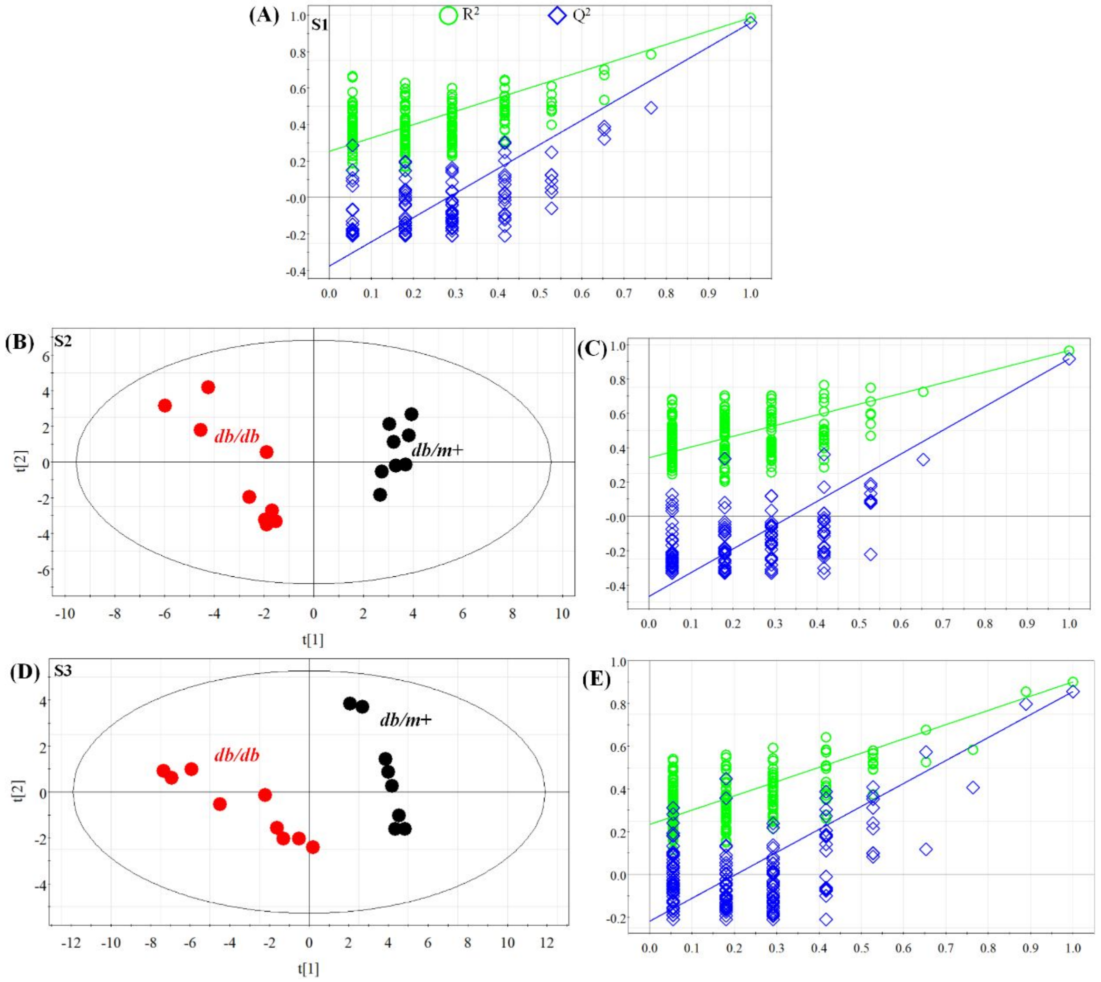

Figure S4 Multivariate statistical analysis based on detected N-acyl glycines in urine samples.

(A) Validation plot of Figure 3b, $R^2X = 0.771$, $R^2Y = 0.995$,$Q^2 = 0.957$. (B) and (D), PLS-DA score plots of N-acyl glycines of *db/m+* (Black dots (●)) and *db/db* (red dots (●)) mice in urine samples at the age of 11-weeks old and 15-weeks old, respectively. (C), Validation plot of (b), $R^2X = 0.841$, $R^2Y = 0.965$,$Q^2 = 0.917$. (E) Validation plot of (D), $R^2X = 0.833$, $R^2Y = 0.9$, $Q^2 = 0.856$.

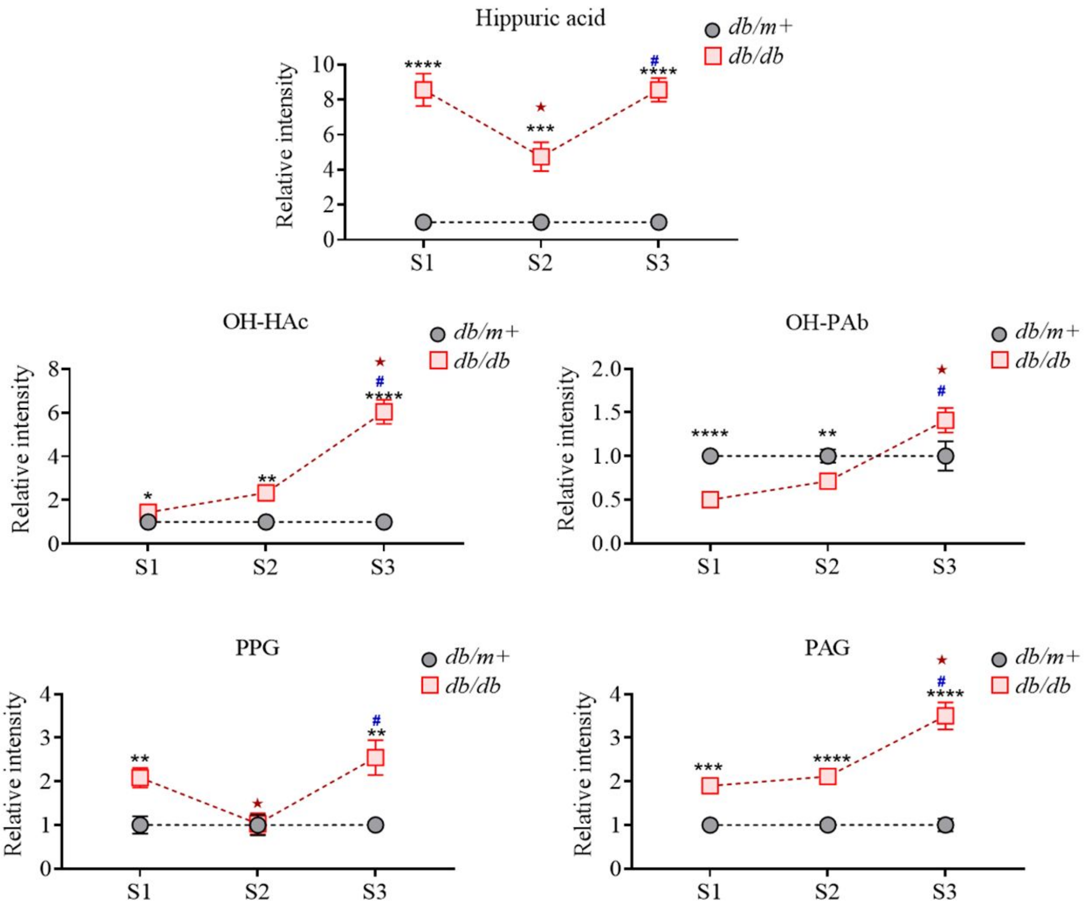

Figure S5 Changes of phenyl derivative-conjugated glycines in urine samples.

S1, stage 1, 7-weeks old. S2, stage 2, 11-weeks old. S3, stage 3, 15-weeks old. *, $p < 0.05$, **, $p < 0.01$, ***, $p < 0.001$, ****, $p < 0.0001$, *db/db* vs *db/m+* from the same stage. Data were analyzed by student t-test. #, $p < 0.05$, normalized *db/db* data (normalized to the control group of the same stage) in S2/S3 compared with S1 ☆, $p < 0.05$, normalized *db/db* data (normalized to the control group of the same stage) in S3 compared with S2, data were analyzed by ANOVA (followed by Tukey's multiple comparison test) method. All data were presented as mean ± se.

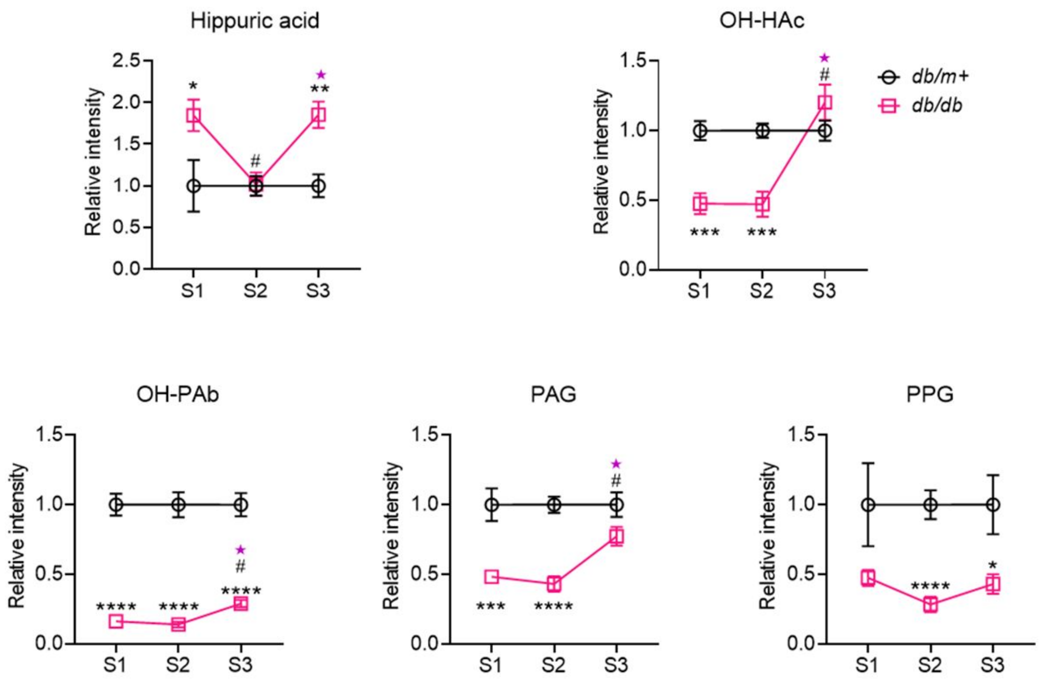

Figure S6 Changes of phenyl derivative-conjugated glycines in urine.
The data were normalized by urinary volume. S1, stage 1, 7-weeks old. S2, stage 2, 11-weeks old. S3, stage 3, 15-weeks old. *, $p < 0.05$, **, $p < 0.01$, ***, $p < 0.001$, ****, $p < 0.0001$, *db/db* vs *db/m+,* student t-test. #, $p < 0.05$, normalized *db/db* data (normalized to the control group of the same stage) in S2/S3 compared with S1 ☆, $p < 0.05$, normalized *db/db* data (normalized to the control group of the same stage) in S3 compared with S2, data were analyzed by ANOVA (followed by Tukey's multiple comparison test) method. All data were presented as mean ± se.
